# Risk of severe COVID-19 from the Delta and Omicron variants in relation to vaccination status, sex, age and comorbidities – surveillance results from southern Sweden, July 2021 to January 2022

**DOI:** 10.2807/1560-7917.ES.2022.27.9.2200121

**Published:** 2022-03-03

**Authors:** Fredrik Kahn, Carl Bonander, Mahnaz Moghaddassi, Magnus Rasmussen, Ulf Malmqvist, Malin Inghammar, Jonas Björk

**Affiliations:** 1 Department of Clinical Sciences Lund, Section for Infection Medicine, Skåne University Hospital, Lund University, Lund, Sweden; 2 School of Public Health and Community Medicine, Institute of Medicine, University of Gothenburg, Gothenburg, Sweden; 3 Social Medicine and Global Health, Department of Clinical Sciences Malmö, Lund University, Malmö, Sweden; 4 Clinical Studies Sweden, Forum South, Skåne University Hospital, Lund, Sweden; 5 Division of Occupational and Environmental Medicine, Lund University, Lund, Sweden

**Keywords:** SARS-CoV-2 vaccine effectiveness, epidemiological surveillance, variant of concern

## Abstract

We compared the risk of severe COVID-19 during two periods 2021 and 2022 when Delta and Omicron, respectively, were the dominating virus variants in Scania county, Sweden. We adjusted for differences in sex, age, comorbidities, prior infection and vaccination. Risk of severe disease from Omicron was markedly lower among vaccinated cases. It was also lower among the unvaccinated but remained high (> 5%) for older people and middle-aged men with two or more comorbidities. Efforts to increase vaccination uptake should continue.

The severe acute respiratory syndrome coronavirus 2 (SARS-CoV-2) variant of concern (VOC) Omicron (Phylogenetic Assignment of Named Global Outbreak (Pango) lineage designation B.1.1.529) was first reported in South Africa on 24 November 2021 and has since spread rapidly worldwide. In a comparison of secondary attack rates within Danish households protection against infection among vaccinated persons was lower if the index case was infected with Omicron rather than Delta (B.1.617.2) [1]. Similarly, a Canadian study, using a test-negative design, found that two doses of a COVID-19 mRNA vaccine did not protect against Omicron infection, while a third dose provided some immediate protection but still substantially less than against Delta [2]. However, available evidence suggests that COVID-19 vaccination still provides high protection against severe disease from Omicron [3-5]. A Danish cohort study among infected individuals found a substantially lower risk of hospitalisation after Omicron infection compared with Delta among both vaccinated and unvaccinated individuals [4]. A limitation in that study was that it included few infections in older age groups, especially among the unvaccinated. To what extent the Omicron variant leads to severe disease in unvaccinated populations, older persons and people with underlying conditions thus merits further investigation. 

The aim of the present study was to monitor Omicron risks in risk groups defined by sex, age and comorbidities in addition to vaccination status. The study was conducted in Scania, southern Sweden, an ethnically and socioeconomically diverse region exceeding 1.3 million inhabitants and with local variation in vaccination coverage between 40% and 90% among adults.

## Study design and data extraction

The study cohort included all persons residing in Scania (Skåne), southern Sweden, on 27 December 2020 (baseline) when vaccinations started (n = 1,384,531) [6,7] and followed them until the date of data extraction (25 January 2022) for hospitalisations and assessment of disease severity. We restricted all analyses to infections that occurred until 9 January 2022 to limit the influence of the time lag from infection to hospitalisation and severe disease. Individuals who died or moved away from the region were censored on the date of death or relocation. The different data sources used in the present study were linked using the personal identification number assigned to all Swedish residents [8]. Regional health registers were used for data on comorbidities, defined as diagnoses in inpatient or specialised care at any time point during the 5 years before baseline in the following disease groups (see Supplementary Table S1 for a detailed list): cardiovascular diseases, diabetes or obesity, kidney or liver diseases, respiratory diseases, neurological diseases, cancer or immunosuppressed states, and other conditions and diseases (Down syndrome, HIV, sickle cell anaemia, drug addiction, thalassaemia or mental health disorder). The number of comorbidities in these groupings was counted and classified as zero, one or at least two in the analyses.

Weekly updates on vaccination date, type of vaccine and dose were obtained from the National Vaccination Register, and data on COVID-19 cases (defined by a positive SARS-CoV-2 test results) from the electronic system SMINet, both kept at the Public Health Agency of Sweden. The regional health registers were accessed continuously to provide data on positive tests rapidly and to assess disease outcomes. Testing was extensive during the study period, and was recommended also for asymptomatic individuals with known or suspected SARS-CoV-2 exposure. Registration in SMINet included positive PCR tests, but also positive rapid antigen tests used in healthcare and at private testing facilities. Self-administrated antigen tests were not registered, but the recommendation to the public was to undergo PCR testing if the self-test was positive. A case was in all analyses defined as a person with a first-time positive test or any positive test for COVID-19 at least 90 days after a prior positive test. Severe COVID-19 was defined as at least 24 h hospitalisation 5 days before until 14 days after a positive test and with a need of oxygen supply ≥ 5 L/min or admittance to an intensive care unit (ICU). Persons who had received at least two COVID-19 vaccine doses, irrespective of vaccine type, more than 7 days before the case date were classified as vaccinated, whereas persons with one or no doses were classified as unvaccinated.

We used data from routine sequencing of samples of infected cases in the Scania region and compared disease risks during three different calendar periods [9]: (i) Delta as the dominating VOC, 2021 week 27–47, (ii) transition period, 2021 week 48–51 when Omicron was first observed, and (iii) Omicron as the dominating VOC, 2021 week 52 (74% sample prevalence) and 2022 week 1 (88% sample prevalence).

## Risk of severe COVID-19

We identified a total of 55,269 coronavirus disease (COVID-19) cases during the three calendar periods of follow-up, of whom 437 (0.79%) were classified as severe. Unvaccinated cases, including those who had received one dose only, were similar with respect to age, sex and comorbidities during the Delta and Omicron periods (Table). Vaccinated cases (persons with 2–3 doses) during the Omicron period were younger and had fewer comorbidities than during the Delta period. 

**Table:** Characteristics of COVID-19 cases, stratified by vaccination status and follow-up period, Scania, Sweden, July 2021–January 2022 (n = 55,269)

	Unvaccinated (0–1 dose; n = 23,217)						Vaccinated (2–3 doses; n = 32,052)					
	Delta 2021 w27–47		Transition 2021 w48–51		Omicron 2021 w52–2022 w1		Delta 2021 w27–47		Transition 2021 w48–51		Omicron 2021 w52–2022 w1	
	%	n	%	n	%	n	%	n	%	n	%	n
Total number	9,680		5,676		7,861		4,031		6,343		21,678	
Severe COVID-19	1.2	118	1.6	90	0.87	68	1.8	71	0.58	37	0.24	53
Age group (years)												
0–17	37.0	3,585	51.4	2,918	39.8	3,126	0.4	18	2.3	144	3.5	756
18–39	43.1	4,169	28.8	1,635	38.4	3,021	26.0	1,047	33.8	2,142	41.6	9,014
40–64	18.6	1,800	17.9	1,016	19.5	1,535	53.4	2,153	53.5	3,393	46.7	10,128
≥ 65	1.3	126	1.9	107	2.3	179	20.2	813	10.5	664	8.2	1,780
Sex												
Female	50.4	4,880	50.0	2,839	51.5	4,049	55.1	2,220	54.2	3,440	54.6	11,833
Male	49.6	4,800	50.0	2,837	48.5	3,812	44.9	1,811	45.8	2,903	45.4	9,845
Comorbidities												
0	87.4	8,465	87.3	4,954	84.0	6,606	71.8	2,896	79.4	5,039	80.4	17,419
1	10.8	1,041	10.5	594	13.1	1,032	18.4	742	15.3	969	14.4	3,120
≥ 2	1.8	174	2.3	128	2.8	223	9.7	393	5.3	335	5.3	1,139
Prior SARS-CoV-2 infection	2.7	265	3.2	180	7.1	562	2.3	94	3.8	238	6.3	1,376
Vaccine doses												
0	85.8	8,306	93.0	5,280	89.0	6,999	NA					
1	14.2	1,374	7.0	396	11.0	862						
2	NA						98.2	3,959	91.7	5,815	83.5	18,103
3							1.8	72	8.3	528	16.5	3,575
Vaccine type^a^												
Comirnaty	NA						79.8	3,218	79.3	5,029	79.2	17,169
Spikevax							6.6	265	9.4	599	11.7	2,533
Vaxzevria							9.5	382	4.9	309	1.2	255
Mixed							4.1	166	6.4	405	7.9	1,721
Time since last dose												
0–3 months	NA						37.1	1,494	14.1	896	21.5	4,651
3–6 months							48.6	1,958	64.4	4,087	58.2	12,608
≥ 6 months							14.4	579	21.4	1,360	20.4	4,419

COVID-19: coronavirus disease; NA: not applicable; SARS-CoV-2: severe acute respiratory syndrome coronavirus 2.

^a^ The following vaccines were used during the study period: Comirnaty (BNT162b2 mRNA, BioNTech-Pfizer, Mainz, Germany/New York, United States), Spikevax (mRNA-1273, Moderna, Cambridge, United States), Vaxzevria (ChAdOx1-S, AstraZeneca, Cambridge, United Kingdom).

Percentages are calculated within each of the six strata.

**Figure 1:**
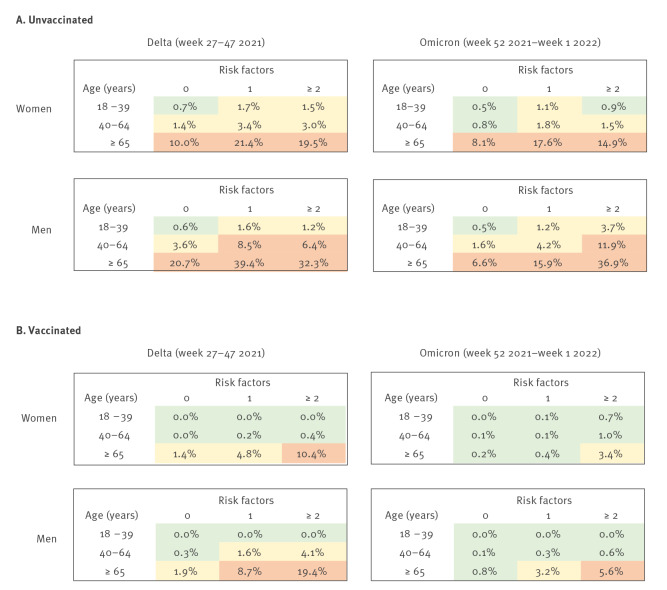
Risk of severe COVID-19 among adult cases without prior infection, stratified by sex, age, number of risk factors (comorbidities), calendar period and vaccination status, Scania, Sweden, July 2021–January 2022 (n = 33,630)

COVID-19: coronavirus disease; SARS-CoV-2: severe acute respiratory syndrome coronavirus 2.

We used logistic regression (Stata SE 14.2, Stata Corp, College Station, Texas, United States) to estimate the effect of the transition from Delta to Omicron on the occurrence of severe disease (see Supplementary Table S2 for the full model results). The analyses were adjusted for sex, age, comorbidities and prior infection, and additionally for booster dose and time since last dose among the vaccinated. The estimated odds of severe COVID-19 were 40% lower (95% confidence interval (CI): 18–56% lower) among unvaccinated and 71% lower (95% CI: 54–82% lower) among vaccinated cases during the Omicron period than during the Delta period. In Figure 1, we present estimates of the absolute risks of severe COVID-19 among cases without prior infection, stratified by sex, age, number of comorbidities, period (Delta, Omicron) and vaccination status (vaccinated, unvaccinated) . The results in Figure 1 are restricted to adults (18 years and older), but the logistic regression analyses used for estimation also included children (see Supplementary Table S3 for the full model results). The risk for severe COVID-19 remained high among unvaccinated, first-time infected cases of both sexes during the Omicron period in the age group 65 years and older, and also among men in the age group 40–64 years with two or more comorbidities (Figure 1A). The risk of severe COVID-19 among vaccinated cases younger than 65 years was low for both sexes during Omicron, even in the presence of comorbidities (Figure 1B). The risk of severe COVID-19 remained elevated among vaccinated cases 65 years and older during Omicron only in the presence of at least one (men) or at least two comorbidities (women).

Green represents risk < 1.0%, yellow 1.0–4.9%, orange ≥ 5.0%.

## Vaccine effectiveness

**Figure 2:**
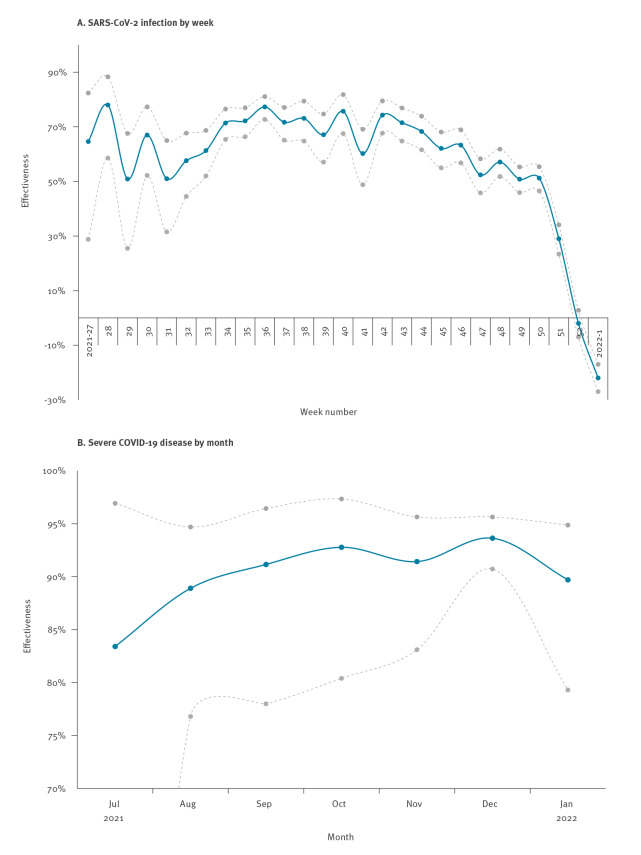
Estimated COVID-19 vaccine effectiveness after at least two doses irrespective of vaccine type, Scania, Sweden, July 2021–January 2022 (n = 55,269 cases; n = 552,690 controls)

We used continuous density case–control sampling [10] nested within the study cohort together with conditional logistic regression to estimate vaccine effectiveness (VE) against infection and severe COVID-19. For each case, 10 controls without a positive test the same week as the case or 90 days prior were randomly selected from the underlying study cohort, matched with respect to sex and age (5-year groups). Median weekly VE against infection was 67% during the Delta period, but showed a declining trend from 2021 week 43 (Figure 2A). A more substantial decline in VE against infection started in the last week of the transition period (2021 week 51) and by end of follow-up, when Omicron dominated, no vaccine protection against infection remained. VE against severe COVID-19 was estimated monthly and remained stable at around 90% across the entire follow-up period irrespective of which VOC dominated (Figure 2B). A separate analysis for the Omicron period, with additional adjustment for comorbidities, confirmed the high protection against severe COVID-19 (VE = 92%; 95% CI: 76–97). Restricting the Omicron period to week 52 in 2021, which reduced misclassification caused by a delay in occurrence and registration of severe cases but potentially increased contamination from remaining Delta cases, only changed this estimate marginally (VE = 91%; 95% CI: 74–97).

Solid lines: vaccine effectiveness; grey dotted lines: 95% confidence intervals.

## Ethical statement

Ethical approval was obtained from the Swedish Ethical Review Authority (2021–00059).

## Discussion

We observed markedly lower risks of severe disease among the vaccinated, i.e. cases who had received at least two doses of a COVID-19 vaccine irrespective of vaccine type, during the period when the SARS-CoV-2 Omicron VOC dominated. The VE thus remained high but changed in nature from protection against both infection and severe disease from Delta to protection only against severe disease from Omicron. The risk of severe disease was also generally lower for unvaccinated cases during the Omicron period, but remained high among older people and middle-aged men with comorbidities. Omicron thus remains a genuine public health concern in countries, populations and subgroups with low vaccination uptake.

The key strengths of our study were the detailed individual-level data on vaccinations, infections and hospitalisations and the possibility to stratify hospitalised people further by disease severity, thereby limiting the misclassification of cases hospitalised *with* rather than *because of* COVID-19. A major limitation was that we lacked data on virus variant for the individual cases in each time period. This is probably less problematic for the reference period where Delta dominated, but it means that some of the cases in the Omicron period were probably infected by Delta. Our estimates may therefore understate the risk reduction associated with Omicron. Another limitation was that testing capacity was under pressure following the surge in cases during the Omicron period, which may have led to overestimation of risks if mild cases were less likely to get tested. It should also be noted that the statistical uncertainty for the estimated VE during the latter part of the Omicron period is substantial, as reflected in the wide confidence interval for January 2022. Continued monitoring of VE during the Omicron period is therefore warranted.

## Conclusion

While the SARS-CoV-2 Omicron VOC rarely leads to severe disease in vaccinated persons, first-time infection can still cause severe disease in unvaccinated persons with advanced age or underlying illnesses. Efforts to increase vaccination uptake across countries, populations and subgroups should thus remain a public health priority.

## Supplementary Data

594000 Supplement
