# Supplementary material for: Risk of severe COVID-19 from the Delta and Omicron variants in relation to vaccination status, sex, age and comorbidities – surveillance results from southern Sweden, July 2021 to January 2022
Source: Euro Surveill. 2022 Mar 3;27(9):2200121. doi: 10.2807/1560-7917.ES.2022.27.9.2200121 (PMC8895467; doi:10.2807/1560-7917.ES.2022.27.9.2200121)
Supplement: Supplement [file 22-00121_BJOERK_Supplement.pdf]

Supplementary Material

Risk of severe COVID-19 from the Delta and Omicron variants in relation to vaccination status, sex, age and comorbidities – surveillance results from southern Sweden, July 2021 – January 2022

Fredrik Kahn[1], Carl Bonander[2], Mahnaz Moghaddassi[3], Magnus Rasmussen[1], Ulf Malmqvist[4], Malin Inghammar[1], Jonas Björk[4,5]

1. Department of Clinical Sciences Lund, Section for Infection Medicine, Skåne University Hospital, Lund University, Lund, Sweden

2. School of Public Health and Community Medicine, Institute of Medicine, University of Gothenburg, Gothenburg, Sweden

3. Social Medicine and Global Health, Department of Clinical Sciences Malmö, Lund University, Malmö, Sweden

4. Clinical Studies Sweden, Forum South, Skåne University Hospital, Lund, Sweden

5. Division of Occupational and Environmental Medicine, Lund University, Lund, Sweden

Content

Supplementary Table S1
Supplementary Table S2
Supplementary Table S3A
Supplementary Table S3B

This supplementary material is hosted by Eurosurveillance as supporting information alongside the article *Risk of severe COVID-19 from the Delta and Omicron variants in relation to vaccination status, sex, age and comorbidities – surveillance results from southern Sweden, July 2021 – January 2022*, on behalf of the authors, who remain responsible for the accuracy and appropriateness of the content. The same standards for ethics, copyright, attributions and permissions as for the article apply. Supplements are not edited by Eurosurveillance and the journal is not responsible for the maintenance of any links or email addresses provided therein.

Supplementary Table S1. Classification of comorbidities.

**Disease group**	**ICD-10 codes (incl KVÅ-codes[a])**
Cardiovascular diseases	I10-I15, I20-I25, I42-I43<br>I50, I60-I69<br>J81
Diabetes or obesity	E10, E11, E66
Kidney or liver diseases	K70.X, K74.3-K74.6, K75.4, K76.0<br>N18.5, N18.9<br>DR016, DR024
Respiratory diseases	A15-A19<br>E84<br>I26, I27<br>J42, J43, J44, J45, J47, J84<br>J96, J98.2, J98.3
Neurological diseases (including dementia)	G00-G99<br>F00-F03
Cancer or immunosuppressed state (including organ transplantation)	C00-C99<br>KAS, FQA, FQB, JJC, GDG, JLE<br>DR046, DR047, DR048<br>D80.0-D80.1<br>D80.5, D81, D82, D83
Other conditions and diseases<br>• HIV<br>• Thalassemia<br>• Sickle cell<br>• Mood disorders<br>• Schizophrenia spectrum disorders<br>• Substance use disorders<br>• Downs syndrome	B20-B24<br>D56, D57<br>F10-F19, F30-F39, F20-F29<br>Q90

[a] Swedish classification of certain interventions during health care visits

Supplementary Table S2. Logistic regression analysis for the effect of calendar period on the odds of severe COVID-19 disease, separate among unvaccinated and vaccinated cases and with adjustment for age, sex, comorbidities, prior infection, time since last dose and booster dose.

	Unvaccinated (0–1 dose) n = 23,217	Vaccinated (2–3 doses) n = 32,052
	OR (95% CI)	OR (95% CI)
Background odds	0.0098 (0.0073 – 0.013)	0.00084 (0.00029 – 0.0025)
Calendar period		
Delta, 2021 w27–47	Ref.	Ref.
Transition, 2021 w48–51	1.3 (1.0 – 1.8)	0.58 (0.37 – 0.91)
Omicron, 2021 w52–2022 w1	0.60 (0.44 – 0.82)	0.29 (0.18 – 0.46)
Age, years		
0 – 17	0.11 (0.06 – 0.23)	0.00[a]
18 – 39	1.0 (Ref.)	1.0 (Ref.)
40 – 64	3.5 (2.6 – 4.8)	3.5 (1.3 – 8.9)
≥ 65	20 (14 – 31)	20 (7.6 – 52)
Sex		
Females	0.58 (0.45 – 0.74)	0.55 (0.39 – 0.77)
Males	Ref.	Ref.
Comorbidities		
0	Ref.	Ref.
1	2.0 (1.4 – 2.7)	4.8 (2.9 – 8.1)
≥2	2.8 (1.8 – 4.2)	14 (8.3 – 23)
Prior SARS-CoV-2 infection	0.15 (0.04 – 0.62)	1.3 (0.57 – 3.1)
Time since last dose	-	
0 – 3 months		Ref.
3 – 6 months		0.94 (0.51 – 1.7)
≥ 6 months		1.3 (0.69 – 2.5)
Booster dose	-	0.84 (0.41 – 1.7)

[a] No severe cases were observed in this group, and 95% CI could therefore not be calculated

Supplementary Table S3A. Logistic regression analysis for the odds of severe COVID-19 disease among unvaccinated (0–1 dose) cases without prior infection, stratified by sex and calendar period (Delta 2021 week 27–47 vs. Omicron 2021 week 52–2022 week 1).

	Females, unvaccinated		Males, unvaccinated	
	Delta period n = 4,722	Omicron period n = 3,727	Delta period n = 4,693	Omicron period n = 3,572
	OR (95% CI)	OR (95% CI)	OR (95% CI)	OR (95% CI)
Background odds	0.0071 (0.0043 – 0.012)	0.0047 (0.0023 – 0.0099)	0.0064 (0.0038 – 0.011)	0.0047 (0.0022 – 0.0098)
Age, years				
0 – 17	0.28 (0.096 – 0.85)	0.00[a]	0.15 (0.03 – 0.64)	0.21 (0.04 – 0.99)
18 – 39	1.0 (Ref.)	1.0 (Ref.)	1.0 (Ref.)	1.0 (Ref.)
40 – 64	2.0 (0.99 – 4.0)	1.6 (0.59 – 4.5)	5.8 (3.1 – 11)	3.5 (1.5 – 8.3)
≥ 65	16 (6.3 – 39)	19 (6.6 – 53)	41 (18 – 92)	15 (5.5 – 41)
Comorbidities				
0	1.0 (Ref.)	1.0 (Ref.)	1.0 (Ref.)	1.0 (Ref.)
1	2.5 (1.2 – 5.0)	2.4 (0.96 – 6.2)	2.5 (1.4 – 4.4)	2.7 (1.2 – 6.1)
≥ 2	2.2 (0.65 – 7.3)	2.0 (0.62 – 6.5)	1.8 (0.70 – 4.7)	8.3 (3.4 – 20)

[a] No severe cases were observed in this group, and 95% CI could therefore not be calculated

Supplementary Table S3B. Logistic regression analysis for the odds of severe COVID-19 disease among vaccinated (2–3 doses) cases without prior infection, stratified by sex and calendar period (Delta 2021 week 27–47 vs. Omicron 2021 week 52–2022 week 1).

	Females, vaccinated		Males, vaccinated	
	Delta period n = 2,165	Omicron period n = 10,976	Delta period n = 1,772	Omicron period n = 9,326
	OR (95% CI)	OR (95% CI)	OR (95% CI)	OR (95% CI)
Background odds	0.00049 (0.000060 – 0.00039	0.00058 (0.00020 – 0.0017)	0.0034 (0.0013 – 0.0090)	0.00090 (0.00037 – 0.0022)
Age, years				
0 – 17	0.00[a]	0.00[a]	0.00[a]	0.00[a]
18 – 39	0.00[a]	0.77 (0.18 – 3.3)	0.00[a]	0.00[a]
40 – 64	1.0 (Ref.)	1.0 (Ref.)	1.0 (Ref.)	1.0 (Ref.)
≥ 65	29 (3.7 – 225)	3.6 (1.1 – 12)	5.7 (2.5 – 13)	9.5 (3.8 – 24)
Comorbidities				
0	1.0 (Ref.)	1.0 (Ref.)	1.0 (Ref.)	1.0 (Ref.)
1	3.6 (0.88 – 15)	1.7 (0.32 – 9.0)	4.9 (1.7 – 14)	3.9 (1.4 -11)
≥ 2	8.3 (2.2 – 31)	17 (4.8 – 58)	12 (4.5 – 34)	6.9 (2.4 – 19)

[a] No severe cases were observed in this group, and 95% CI could therefore not be calculated
